# Autonomous Nanorobots as Miniaturized Surgeons for Intracellular Applications

**DOI:** 10.3390/nano14070595

**Published:** 2024-03-28

**Authors:** Daitian Tang, Xiqi Peng, Song Wu, Songsong Tang

**Affiliations:** 1 Luohu Clinical Institute, School of Medicine, Shantou University, Shantou 515000, China; 21dttang@stu.edu.cn (D.T.); 13xqpeng@stu.edu.cn (X.P.); 2 Andrew and Peggy Cherng Department of Medical Engineering, California Institute of Technology, Pasadena, CA 91125, USA

**Keywords:** nanorobots, robust and controlled propulsion, enhanced intracellular delivery, intracellular biosensing, organelle targeting

## Abstract

Artificial nanorobots have emerged as promising tools for a wide range of biomedical applications, including biosensing, detoxification, and drug delivery. Their unique ability to navigate confined spaces with precise control extends their operational scope to the cellular or subcellular level. By combining tailored surface functionality and propulsion mechanisms, nanorobots demonstrate rapid penetration of cell membranes and efficient internalization, enhancing intracellular delivery capabilities. Moreover, their robust motion within cells enables targeted interactions with intracellular components, such as proteins, molecules, and organelles, leading to superior performance in intracellular biosensing and organelle-targeted cargo delivery. Consequently, nanorobots hold significant potential as miniaturized surgeons capable of directly modulating cellular dynamics and combating metastasis, thereby maximizing therapeutic outcomes for precision therapy. In this review, we provide an overview of the propulsion modes of nanorobots and discuss essential factors to harness propulsive energy from the local environment or external power sources, including structure, material, and engine selection. We then discuss key advancements in nanorobot technology for various intracellular applications. Finally, we address important considerations for future nanorobot design to facilitate their translation into clinical practice and unlock their full potential in biomedical research and healthcare.

## 1. Introduction

In his seminal 1959 speech titled “There’s Plenty of Room at the Bottom”, physicist Richard Feynman envisioned the manipulation of matter at atomic and molecular scales. His ideas of ‘swallowing the surgeon’ inspired the development of tiny surgical robots designed to be ingested [1,2]. These visionary concepts have challenged scientists to explore the possibilities of fabricating nanoscale machines capable of controlled operation within the human body [3,4,5]. However, the constraints imposed by the nanoscale limit the incorporation of traditional electronic power and control systems into nanomachines. Generating propulsive force at low Reynolds numbers and overcoming Brownian motion represent primary obstacles in designing untethered nanorobots [6,7]. The rapid evolution of nanotechnology has facilitated the emergence and advancement of nanorobots with various designs and propulsion mechanisms.

Nanorobots are miniaturized machines at nanoscale that are capable of converting local energy or external power to propulsive force for achieving effective propulsion [8,9,10,11]. These nanorobots typically have a diameter of less than 1 μm with various shapes, such as rod, sphere, helical, hollow, or other complex structures. The material composition may vary for different applications, including rigid metals, biocompatible polymers, and 3D-printed resin [5,12,13]. Extensive efforts have been dedicated to developing nanorobots with various propulsion modes, including chemical propulsion [9,14,15,16,17], magnetic propulsion [4,18,19,20,21], ultrasound propulsion [22,23,24], and light propulsion [25,26,27,28]. These autonomous tiny machines enable controlled operations in narrow spaces or hard-to-reach sites to accomplish complicated tasks [29,30,31,32]. These advancements have endowed nanorobots with superior performance compared to traditional passive systems in biomedical domains, such as biosensing, detoxification, and drug delivery [30,33,34,35]. As the demand for precision therapy has prompted a shift in therapeutic targeting from tissues to individual cells, the aim is to understand cellular mechanisms underlying diseases and achieve enhanced therapeutic efficacy [32,36,37]. Autonomous nanorobots represent an attractive platform for overcoming the constraints of traditional passive systems.

Passive systems utilized for intracellular applications mainly rely on diffusion-based transport, encountering several challenges that impede their effectiveness [38]. For instance, diffusion-based delivery commonly takes a longer time to reach the cell, leading to low delivery efficiency and inadequate targeting. Passive particles struggle to rapidly pass the biological barrier of the cell membrane due to the deficiency of external power forces, thereby limiting the cellular internalization of loaded cargo. In contrast, nanorobots capable of effective and steerable motion enable active and targeted seeking of the desired cells [39,40,41,42]. Their capability of harnessing energy from the surrounding solution and an external power source endows nanorobots with sufficient force to rapidly penetrate the cell membrane [43,44]. The internalized nanorobots can leverage their controlled propulsion to rapidly interact with targeted proteins, molecules, and organelles for advanced intracellular biosensing and drug delivery. Nanorobots act as miniaturized intracellular surgeons to directly regulate cellular metabolism with minimal invasion. This advanced nanomachine capable of cellular or subcellular access introduces next-generation robotic medical devices for precision therapy, holding great potential for achieving maximized therapeutic outcomes with minimized toxicity and healthcare costs.

**Figure 1:**
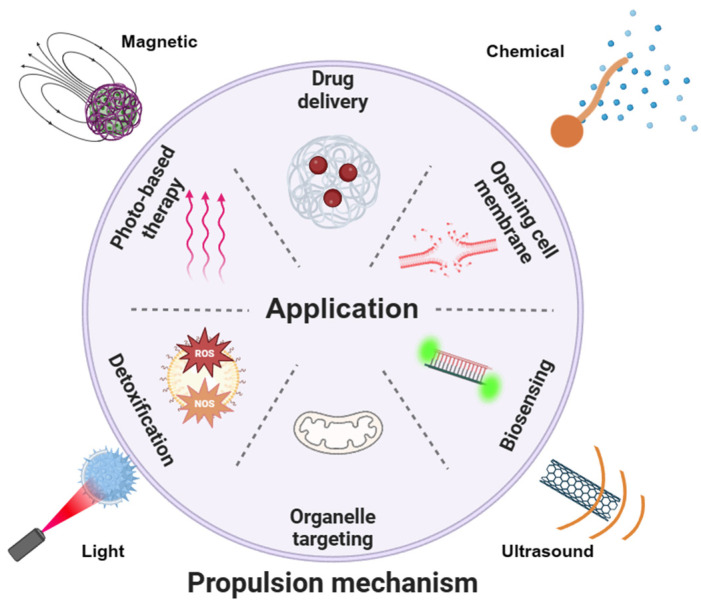
Schematic of various propulsion mechanisms and intracellular applications of nanorobots. Various propulsion modes may be suitable for desired applications.

In this review, we will first discuss the propulsion modes of nanorobots. The material selection, structure design, and propulsion mechanisms will be discussed in each propulsion mode (Figure 1). Then, we will review the reported intracellular applications of nanorobots, including opening cell membranes, biosensing, detoxification, photo-based therapy, drug delivery, and organelle targeting. We will provide detailed insights into the construction of nanorobots, including surface modification techniques, and discuss the enhancements enabled by nanorobots. Table 1 summarizes the representative examples of nanorobots for intracellular applications. We will highlight various studies to describe how the effective propulsion of nanorobots results in superior performance compared to static counterparts. Finally, we will outline three key considerations regarding nanorobotic designs to facilitate future advancements toward clinical trials.

## 2. Propulsion Mechanisms

Tremendous efforts have been devoted to developing nanorobots with various propulsive engines. Breaking the geometric symmetry of robotic design is essential to achieve directional and sufficient propulsive force [15,74,75]. Here, we mainly focus on four propulsion modes: chemical propulsion, magnetic propulsion, ultrasound propulsion, and light propulsion. The motion mechanism and required robotic building entities will be discussed.

### 2.1. Chemical Propulsion

**Figure 2:**
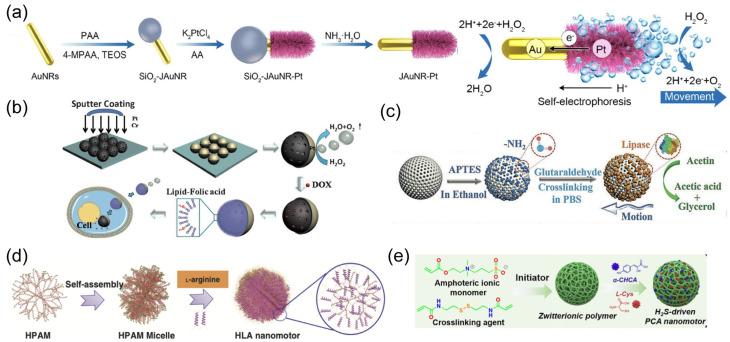
(**a**) Schematic illustration of the preparation and movement of a JAuNR-Pt nanorobot. Reprinted with permission from [79]. Copyright © 2022, American Chemical Society. (**b**) Synthetic procedure for Janus mesoporous silica nanoparticle (MSN) nanorobots, as well as subsequent drug loading, lipid bilayer functionalization, transportation, and drug release. Reprinted with permission from [34]. Copyright © 2014, John Wiley and Sons. (**c**) Schematic representation of the preparation of lipase-powered nanorobots, whose motion is triggered by the catalytic decomposition of triacetin. Reprinted with permission from [80]. Copyright © 2019, John Wiley and Sons. (**d**) Schematic illustration of the preparation of a zwitterion-based nanomotor. Reprinted with permission from [81]. Copyright^©^ 2019, Springer Nature. (**e**) Illustration of the synthetic process of a PCA nanorobot. Reprinted with permission from [82]. Copyright © 2021, John Wiley and Sons.

The chemical engines of nanorobots operate through chemical reactions between the catalytic robotic body and the surrounding solution, converting chemical energy into an effective driving force [16,76]. Noble metals have been widely adopted as nanorobotic bodies in earlier studies, such as platinum [77] and silver [78]. Correspondingly, hydrogen peroxide (H_2_O_2_) is commonly employed as the propulsion fuel. An oxygen molecule is generated in the catalytic reaction rather than an oxygen bubble due to the insufficient nucleation site at nanoscale, whereas other reaction products may vary depending on the robotic compositions. For example, the oxidation electrochemical half-reaction occurs on the Pt segment of bimetallic Pt/Au nanowire robots, producing hydrogen ions (H^+^) on that side [79]. The electrokinetic flow of H^+^ toward the Au side for reduction half-reaction generates an electrophoretic gradient to propel bimetallic nanorobots (Figure 2a). The catalytic process can be altered by combining noble metals with inorganic entities. Taking the Janus Pt/silica nanospheric robots as an example [34], H_2_O_2_ is decomposed on the asymmetrically coated Pt side, yielding water and oxygen molecules (Figure 2b). The uneven distribution of reaction products on the nanorobot surface induces a directional flow toward lower concentration, creating self-diffusiophoretic locomotion for nanorobots.

The increasing demand from the biomedical community has led to the expansion of catalytic engines beyond noble metals to include porous metal–organic frameworks (MOFs) [83], polymers [84], and enzymes (e.g., catalase, urease, lipase) (Figure 2c) [80,85,86]. Considering the potential toxicity of H_2_O_2_ fuel, catalytic systems that can utilize bioavailable fuels are highly preferable. An endogenous enzyme capable of biocatalytic decomposition of bioavailable fuel represents an attractive option for improving the biocompatibility and adaptability of nanorobots in the biomedical field [87]. The enzyme engines [88], such as urease or glucose oxidase (Gox), have been verified to provide an effective driving force for nanorobots in the presence of biofluids containing urea or glucose, respectively [89].

The intracellular metabolic pathways also inspire the design of chemical-powered nanorobots propelled by endogenous substrates. A typical metabolic pathway in mammalian cells involves the conversion of L-arginine into nitric oxide (NO) in the presence of nitric oxide synthetases (NOSs) or reactive oxygen species (ROS) [90]. By leveraging this biocatalytic process, Mao et al. designed hyperbranched polyamide-based nanorobots with L-arginine coating (Figure 2d) [81]. L-arginine serves as propulsion fuel that can be converted into NO in response to ROS, generating effective locomotion for nanorobots. Additionally, L-cysteine is utilized as an enzyme-responsive fuel to construct self-powered nanorobots based on zwitterionic polymers (Figure 2e) [82]. The overexpressed cystathionine b-synthase (CBS) inside tumor cells enables the decomposition of L-cysteine into hydrogen sulfide (H_2_S), inducing motion for nanorobots in the tumor microenvironment.

### 2.2. Ultrasound Propulsion

**Figure 3:**
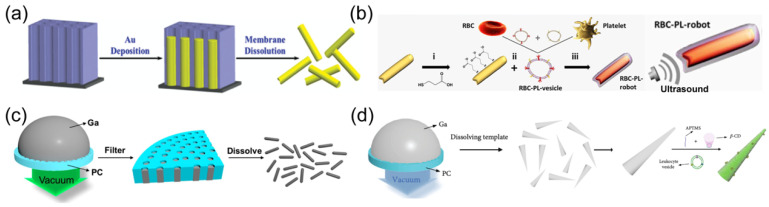
(**a**) Synthesis of Au nanorod robots by membrane-template electrodeposition method. Reprinted with permission from [93]. Copyright © 2014, John Wiley and Sons. (**b**) Schematic of ultrasound-powered Au nanorobots modified with carboxyl group to facilitate the cell membrane coating for biodetoxification. Reprinted with permission from [94]. Copyright © 2018, American Association for the Advancement of Science. (**c**) Scheme for the preparation of liquid metal gallium nanorobots by the pressure-filter-template method. Reprinted with permission from [50]. Copyright © 2018, American Chemical Society. (**d**) Schematic of fabrication and surface modification by aminopropyltrimethoxysilane (APTMS) of the liquid metal gallium nanorobots. Reprinted with permission from [95]. Copyright © 2018, American Association for the Advancement of Science.

Ultrasound waves offer deep tissue penetration without causing damage [91]. This non-invasive power with remote control has been widely applied in medical and clinical fields [33]. As an external power source of nanorobots, an acoustic streaming force is generated in the acoustic field to propel these tiny machines [92]. The most frequently used building entity is a Au nanorod with a concave end fabricated using a membrane-template electrodeposition method [93]. Au is first deposited within the cylindrical nanopore of a polycarbonate (PC) membrane. Subsequently, the membrane template is dissolved to release fabricated Au nanowires with concave ends (Figure 3a). The surface modification of rod-like Au nanorobots can be facilely realized by reacting with thiol (-SH)-functionalized chemical groups, such as amino or carboxyl, expanding their versatility for broader adaptions in biomedical domains. For example, Au nanorobots were modified with a carboxyl group to facilitate the cell membrane coating for biodetoxification (Figure 3b) [93,94].

The robotic composition constructed through template-assisted electrodeposition protocol can be readily expanded to other materials. The layer-by-layer (LBL) technology was used to deposit poly(styrenesulfonate) (PSS) and poly (allylamine hydrochloride) (PAH) into conical nanopores of a PC membrane. After dissolving the template, polymer-based nanorobots with tubular cone shapes were formed, which could be propelled with a small opening leading orientation upon the acoustic field. This template method was slightly modified to deposit liquid metals, which have attracted significant interest in biomedicine and soft electronics due to their low toxicity and superior fluidity. A droplet of liquid metal, such as gallium, was first added to the surface of a porous PC membrane. Afterward, pressure filtration was applied to squeeze liquid metal into nanopores, yielding rod-like nanorobots (Figure 3c) [50]. Such liquid metal-based nanorobots enabled the surface modification with aminopropyltrimethoxysilane (APTMS) to load the anticancer drug carbonylated β-cyclodextrin (β-CD) (Figure 3d) [95]. The leukocyte membrane can be further fused on the shell of liquid metal nanorobots to improve their biocompatibility and cancer-targeting capability.

### 2.3. Magnetic Propulsion

One crucial aspect of achieving magnetic propulsion is the integration of magnetic materials, such as iron (Fe), nickel (Ni), cobalt (Co), or iron oxide (Fe_3_O_4_), into the robotic body [96]. External magnetic fields can propel and navigate nanorobots along a predefined path [97]. This fuel-free propulsion with remote maneuverability allows precision control of motile nanorobots to fulfill various missions in complex and dynamic environments [98]. The robotic structure design and motion mechanism are inspired by bacterial movement, which relies on flagella rotation to propel them forward and backward [99]. A rotating magnetic field is generated by an external actuation system to mimic the function of rotating flagella. A typical magnetic actuation system consists of a power supply, data acquisition controller, and magnetic components, which can be permanent magnets or electromagnets [100].

**Figure 4:**
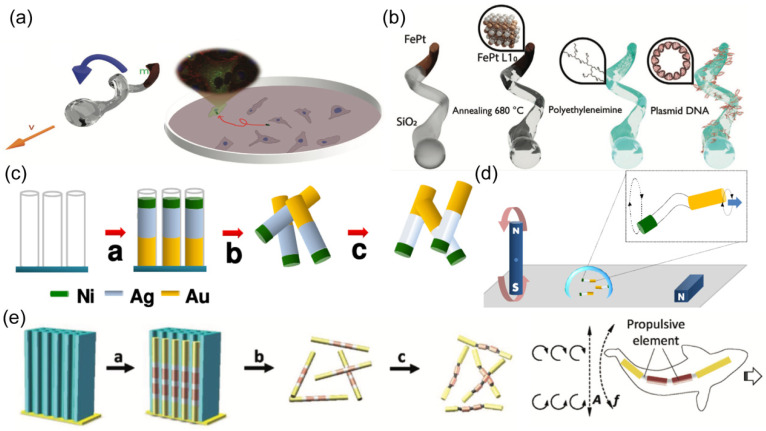
(**a**) Schematic of helical nanorobots capable of corkscrew-like motion. Reprinted with permission from [103]. Copyright © 2020, John Wiley and Sons. (**b**) Schematic of a helical nanorobot coated with Fe and Pt alloy. Reprinted with permission from [103]. Copyright © 2020, John Wiley and Sons. (**c**) Preparation of the Au/Ag/Ni nanowire robot by template electrodeposition. Reprinted with permission from [104]. Copyright © 2018, American Chemical Society. (**d**) Schematic of the motion of a three-segment nanowire robot in a rotating magnetic field. Reprinted with permission from [104]. Copyright © 2018, American Chemical Society. (**e**) Schematic of the fabrication process of a fish-like nanorobot through the sequential electrodeposition of gold, silver, nickel, silver, nickel, silver, and gold, and the propulsion mechanism of the nanorobot in an oscillating magnetic field. Reprinted with permission from [105]. Copyright © 2016, John Wiley and Sons.

The structure design of nanorobots may vary from different motion mechanisms. The corkscrew-like motion requires a helical structure. The rotation along the helical axis can be transformed into nonreciprocal translational locomotion (Figure 4a). A physical vapor deposition approach named glancing angle deposition (GLAD) is proposed to fabricate helical nanostructures with spherical heads and helical tails [101,102,103]. Then, the magnetic layer was coated on the helical structure using techniques such as sputtering or e-beam. To enhance the biocompatibility of magnetic materials, iron and platinum were co-deposited, followed by an annealing process (Figure 4b) [103]. The resulting FePt helical nanorobots exhibited superior biosafety when incubated with living cells.

The surface workers with flexible tails have also been designed to achieve robust propulsion in response to an external magnetic field. Gao et al. designed a three-segment nanowire robot composed of Au, Ag, and Ni prepared by template electrodeposition (Figure 4c) [104]. The central Ag part was partially dissolved by H_2_O_2_, yielding a flexible joint between the Au ‘head’ and the Ni ‘tail’. The magnetic Ni tail rotated upon an external rotating magnetic field, resulting in the rotation of the Au part with different amplitude. The broken systemic symmetry induced effective motion for nanorobots (Figure 4d). Another work reported a fish-like nanorobot with multi-segments, fabricated by sequential electrodeposition of gold, silver, nickel, silver, nickel, silver, and gold (Figure 4e) [105]. The two Au parts served as the head and caudal fin, respectively. The two nickel segments formed the body, connecting to other parts through three flexible porous silver joints. The oscillating magnetic field induced periodical bending of fish-like nanorobots, generating travelling waves to propel nanorobots.

### 2.4. Light Propulsion

Light propulsion offers a fuel-free method for propelling nanorobots, eliminating the need for complex actuation systems and enabling remote spatial control in a non-invasive manner [106]. The photoactive materials integrated with nanorobots respond to light irradiation with different wavelengths coordinated with various motion mechanisms [107].

**Figure 5:**
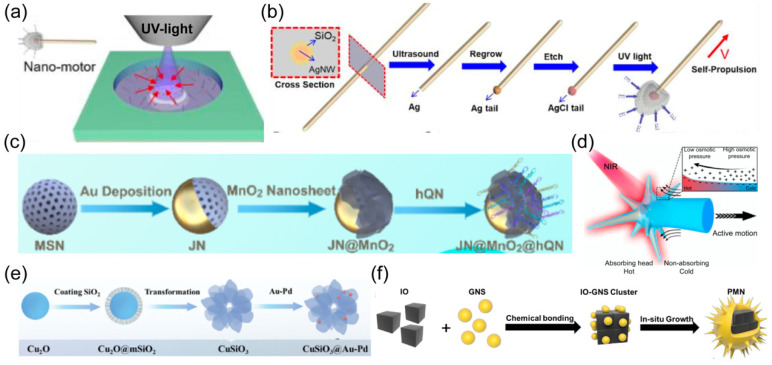
(**a**) Schematic of a light power source for nanorobots. Reprinted with permission from [108]. Copyright © 2019, American Chemical Society. (**b**) Schematic of the fabrication and self-propulsion of match-like nanorobot consisting of silicon dioxide-coated silver nanowires and spherical AgCl tails. Reprinted with permission from [108]. Copyright © 2019, John Wiley and Sons. (**c**) Schematic of nanorobot with unevenly coated Au layer on spherical surface. Reprinted with permission from [54]. Copyright © 2020, American Chemical Society. (**d**) Schematic illustration of NIR propulsion mechanism of urchin-like nanorobot. Reprinted with permission from [67]. Copyright © 2022, Springer Nature. (**e**) Synthesis of CuSiO_3_ flower-like nanorobots. Reprinted with permission from [109]. Copyright © 2024, John Wiley and Sons. (**f**) Schematic of preparation process of photomagnetically powered spiky nanorobots. Reprinted with permission from [110]. Copyright © 2024, John Wiley and Sons.

Photocatalytic materials can be activated by UV light and generate a photochemical reaction [26]. A matchlike nanorobot was designed that consisted of a silica-coated Ag nanowire and a spherical AgCl tail (Figure 5a) [108]. UV light illumination initiated the photocatalytic decomposition of AgCl, generating self-diffusiophoretic propulsion for nanorobots (Figure 5b). However, employing UV light as the power source faces constraints in the biomedical field due to its limited tissue penetration depth and potential damage and toxicity to normal tissue.

Near-infrared (NIR) light has longer wavelengths compared to visible and ultraviolet light, allowing it to penetrate deeper into biological tissues. NIR light also shows good biosafety and biocompatibility with minimal tissue absorption and low phototoxicity [27]. These merits make NIR light particularly well-suited for in vivo applications, enabling the activation and manipulation of nanorobots deep within the body. Photothermal materials are employed to absorb NIR light and convert it into thermal energy. The Janus structure of nanorobots is required to generate an asymmetric thermal gradient, inducing self-thermophoretic motion. The photothermal effect of Au is widely used to prepare NIR-powered nanomachines (Figure 5c). For example, the Au layer was unevenly coated on the surface of spheric [28,54] or urchin-like [67] nanorobots (Figure 5d). Au-Pd nanoalloys were also assembled into flower-like nanorobots to provide a photothermal effect (Figure 5e) [109]. A spiky nanorobot was synthesized by coating Au nanotips onto the magnetic nanoparticle (Figure 5f) [110].

Researchers have also developed alternative materials besides Au to enlarge the options for photothermal engines. Copper sulfide exhibits strong absorption in the NIR-II region with a distinct photothermal feature, which was encapsulated in Janus hydrogel nanorobots to offer propulsive force under the irradiation of NIR-II light [111]. The photothermal effect of carbon, obtained by pyrolysis, was incorporated with a jellyfish-like nanorobot [112]. Organic semiconducting polymer nanoparticles with the capability of light-to-heat converting were also verified to couple with Janus nanorobots, inducing effective propulsion upon NIR irradiation [113].

The vast family of photochemical and photothermal materials enables versatile designs of light-actuated nanorobots. The NIR light with superior biocompatibility and tissue penetration depth is preferable as the remote power source of nanorobots for broader biomedical missions.

## 3. Intracellular Applications

### 3.1. Opening Cell Membrane

The cell membrane, also known as the plasma membrane, serves as a crucial selectively permeable barrier enveloping the cell, separating its internal environment from its external surroundings [114]. This lipid bilayer plays a critical role in maintaining cellular homeostasis by controlling the passage of substances into and out of the cell. Endocytosis controls the cellular internalization of nutrients, signaling molecules, and certain therapeutic agents [115]. However, this natural process tends to be slow due to the involved complex pathways, including fusion with the membrane and escape from lysosomes. It may cause limited bioavailability of therapeutic agents and a longer curative course. By opening or permeabilizing the cell membrane, external cargo is allowed to rapidly enter the cytoplasm to regulate and control cell metabolism, showing promising potential to accelerate disease diagnosis and therapy [116,117,118]. Minimizing cellular damage to maintain cell viability is a critical consideration in this operation.

**Figure 6:**
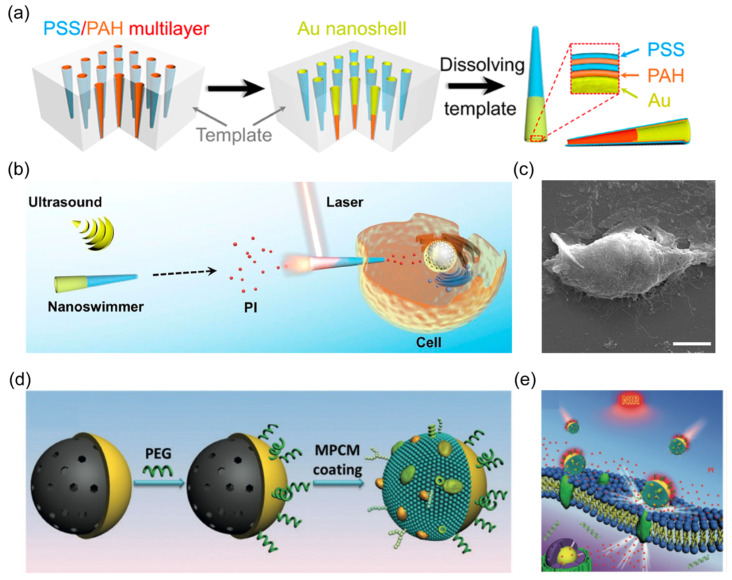
(**a**) Schematic showing fabrication of the AuNS-functionalized tubular polymer nanorobots by LBL technology. Reprinted with permission from [52]. Copyright © 2019, American Chemical Society. (**b**) Schematic of cell poration enabled by acoustic-powered nanorobots with the assistance of NIR light. Reprinted with permission from [52]. Copyright © 2019, American Chemical Society. (**c**) SEM image of the nanorobot after cell poration. Scale bar, 5 μm. Reprinted with permission from [52]. Copyright © 2019, American Chemical Society. (**d**) Schematic of the preparation of Janus mesoporous silica nanorobot with macrophage cell membrane coating. Reprinted with permission from [72]. Copyright © 2019, John Wiley and Sons. (**e**) Schematic of NIR-powered nanorobots for thermomechanically percolating the cell membranes. Reprinted with permission from [72]. Copyright © 2019, John Wiley and Sons.

Autonomous nanorobots with precise manipulation at nanoscale offer an attractive tool to open cell membranes. These tiny machines reduce the contact area with cell membranes, enabling minimized-invasive operation. Although the propulsive force of nanorobots is unable to mechanically open the cell membrane, their capability of incorporating with an external power source offers a feasible route to harvesting sufficient energy to percolate or penetrate the cell membrane. Xuan et al. developed an NIR light-driven Janus mesoporous silica nanorobot with half-coated Au shells and a macrophage cell membrane (Figure 6d) [72]. The photothermal effect of the Janus Au layer generated a heat gradient upon NIR irradiation, leading to self-thermophoretic propulsion for nanorobots. Furthermore, the modification of the macrophage cell membrane improved the specific binding of active nanorobots with cancer cells.

After adhesion with the cancer cell membrane, the photothermal effect of nanorobots induced thermomechanical perforation of the cytomembrane, facilitating rapid internalization of external agents (Figure 6e). In another study, Wang et al. reported a multilayer tubular nanorobot constructed using a gold nanoshell-functionalized polymer (Figure 6a) [52]. The nanorobots exhibited effective motion toward targeted cells under an acoustic field (Figure 6b). When tubular acoustic-powered nanorobots interact with the cell membrane on the side of a small opening (Figure 6c), NIR laser application induced cell membrane poration within 0.1 s. The Au composition of nanorobots generated an instantaneous photothermal effect, which was converted to sufficient photomechanical force to penetrate the cell membrane. These demonstrated in vitro cell membrane opening techniques are poised to provide crucial research support for the intracellular applications of nanorobots in the biomedical domain.

### 3.2. Biosensing

Intracellular biosensing plays a crucial role in understanding cellular dynamics, monitoring cellular responses to various stimuli, and advancing biomedical research and applications. However, traditional passive sensor systems face significant challenges, including slow endocytosis processes and off-targeted probes that fail to enter targeted cells efficiently. To address these issues, steerable nanorobots with abundant surface modification avenues have emerged as promising biosensors [119]. The controllable movement of nanorobots enables active seeking and binding with targeted cells, enhancing their cellular internalization and facilitating rapid detection of intracellular biomarkers [120].

**Figure 7:**
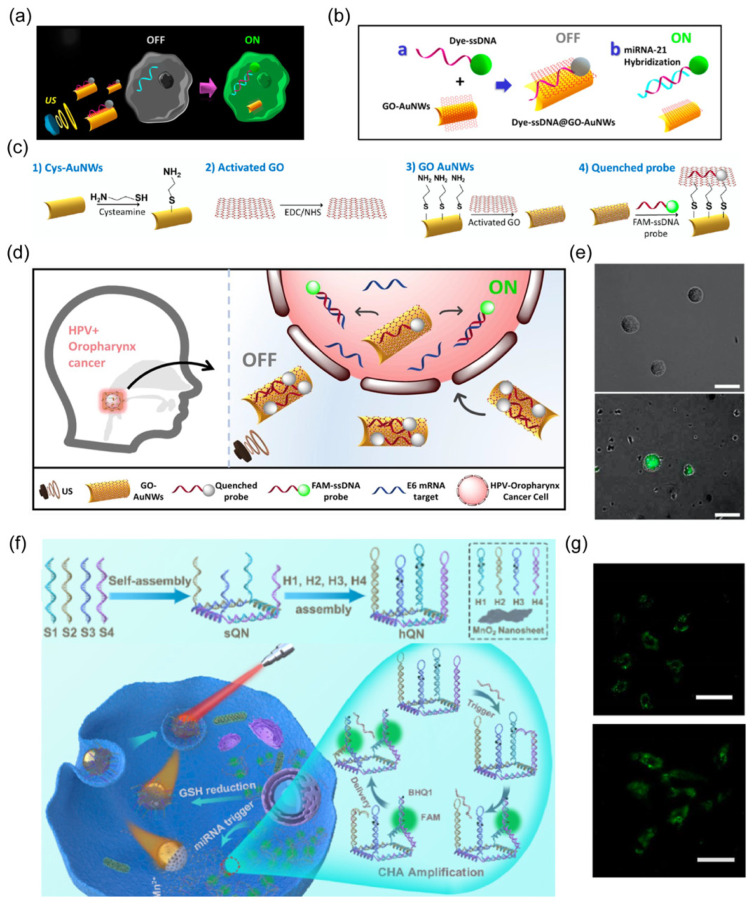
(**a**) Schematic of “OFF-ON” fluorescent switching system of nanorobots for the specific detection of miRNA-21 in cancer cells. Reprinted with permission from [45]. Copyright © 2015, American Chemical Society. (**b**) The functionalized step of ssDNA@GO Au nanorobots. Reprinted with permission from [45]. Copyright © 2015, American Chemical Society. (**c**) Schematic illustrating the loading of probes onto the nanorobot surface. Reprinted with permission from [73]. Copyright © 2019, American Association of Otolaryngology-Head and Neck Surgery Foundation. (**d**) The overall concept of OFF-ON nanorobotic detection system for intracellular HPV16 E6 mRNA transcripts. Reprinted with permission from [73]. Copyright © 2019, American Association of Otolaryngology-Head and Neck Surgery Foundation. (**e**) Fluorescence images of modified nanorobots after 15 min incubation with HPV-negative cells (i) and HPV-positive cells under US field (ii). Scale bar: 25 mm. Reprinted with permission from [73]. Copyright © 2019, American Association of Otolaryngology-Head and Neck Surgery Foundation. (**f**) Illustration of the assembly process of the hQN probe and imaging miRNAs in cells. Reprinted with permission from [28]. Copyright © 2020, American Chemical Society. (**g**) CLSM images of miRNA21 in HepG2 treated with variant nano systems. (i) MnO_2_@hQN, (ii) JN@MnO_2_@hQN nanorobots. Reprinted with permission from [28]. Copyright © 2020, American Chemical Society.

A pioneering study reported the effective motion of Au nanorod robots inside living cells upon an acoustic field without damaging cell viability [121]. This miniaturized vehicle is further developed as an active intracellular biosensor by leveraging the versatile modification methods of the robotic Au surface. Wang’s group designed a microRNA (miRNA) sensor by modifying Au nanorod robots with graphene oxide through covalent binding (Figure 7a) [45]. Then, a dye-labeled specific single-stranded DNA (ssDNA) probe was absorbed on graphene oxide (GO) through π-stacking interaction, where the fluorescence was quenched (Figure 7b).

The preferential binding of ssDNA with targeted miRNA led to detachment from the GO surface and fluorescence recovery. Upon an acoustic field, the rapid movement of nanorobots enables faster cellular internalization of this active sensor, resulting in highly efficient hybridization between ssDNA and targeted miRNA inside cancer cells. The intracellular “OFF-ON” fluorescence switch verified the accelerated biosensing process of Au-based nanorobots. The loaded probe in this system can be readily expanded to other sensor types. A similar approach was subsequently presented to detect mRNA transcripts of human papillomavirus (HPV)-associated oropharyngeal cancer (OPC) by loading dye-labeled ssDNA onto acoustic-powered Au/GO nanorod robots (Figure 7c–e) [73]. Such nanorobots were also surface-modified with fluorescein-labeled DNA aptamers to detect overexpressed AIB1 oncoproteins inside tumor cells [46].

Another external power source, light, is employed to provide robust energy for propelling nanorobotic biosensing platforms inside cells (Figure 7f). Lin et al. developed a Janus nanorobot with asymmetric coating of Au and MnO_2_ nanosheets [28]. An miRNA-responsive probe with hairpin DNA quadrangular nanostructure (hQN) was immobilized on MnO_2_ nanosheets. After entering cells, intracellular glutathione was found to degrade MnO_2_, inducing the release of hQN. The interaction between hQN and targeted miRNA initiated the catalyzed hairpin assembly (CHA), triggering a cascade fluorescence amplification reaction (Figure 7g). Upon NIR irradiation, the nanorobots underwent self-thermophoretic locomotion inside the cell, which enhanced their binding to targeted miRNA with verified stronger fluorescence compared to other static groups.

### 3.3. Detoxification

The intricate cellular metastasis may involve the generation or elimination of toxic substrates derived from both endogenous metabolites and exogenous environmental toxins. This delicate balance is crucial for maintaining cellular health in normal conditions. The overexpression of intracellular toxins may lead to cellular damage and dysfunction, thereby contributing to the development and progression of pathological conditions. For example, excessive reactive oxygen species (ROS) can induce oxidative stress, causing damage to intracellular components and cellular signal dysregulation [122]. The elevated level of ROS has been implicated in various diseases, such as neurodegenerative diseases, cardiovascular diseases, and cancer [123]. Endocytosis of passive agents to alleviate symptoms of toxication usually requires a longer duration, leading to compromised therapeutic efficacy, particularly in cases of urgent or acute intoxication [124]. Therefore, it is highly in demand to develop advanced scavengers capable of rapidly detecting and neutralizing toxic contaminants to restore normal cellular homeostasis.

**Figure 8:**
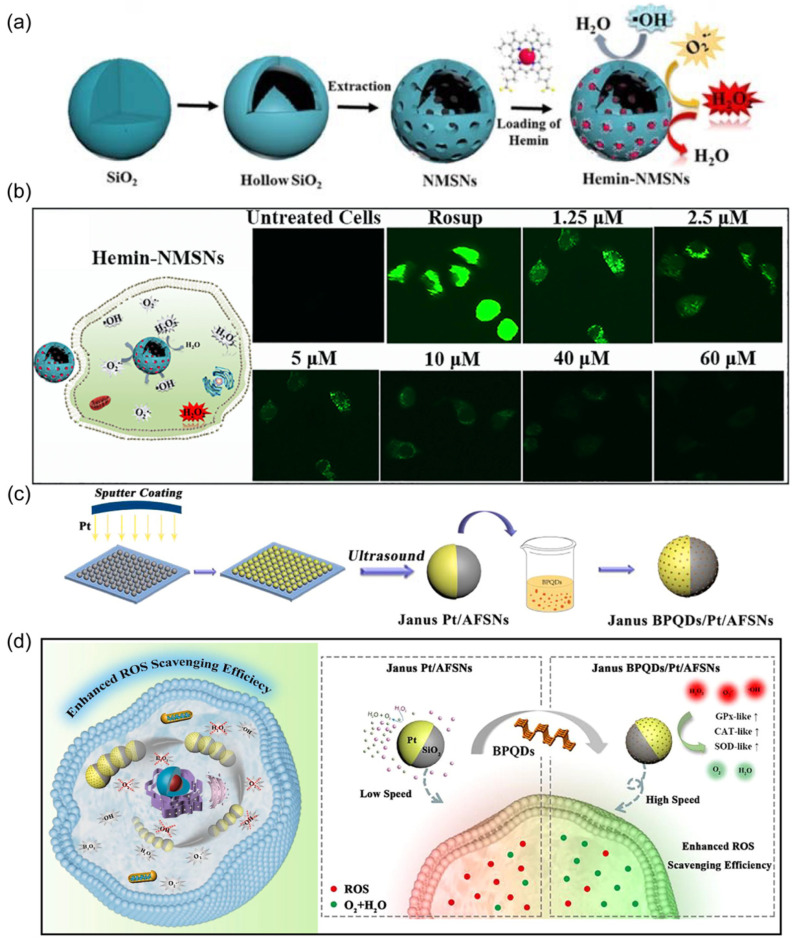
(**a**) Schematic indicating the synthesis of hemin-NMSNs and the subsequent ROS scavenging. Reprinted with permission from [66]. Copyright © 2020, Elsevier. (**b**) Fluorescence images of HUVECs after treatment with hemin-NMSN nanorobots. Reprinted with permission from [66]. Copyright © 2020, Elsevier. (**c**) Synthetic procedure of Janus BPQD/Pt/AFSN nanorobots. Reprinted with permission from [68]. Copyright © 2020, Elsevier. (**d**) Schematic of the intracellular motion of Janus BPQD/Pt/AFSN nanorobots and their application for efficiently scavenging ROS to reduce cellular oxidative stress. Reprinted with permission from [68]. Copyright © 2020, Elsevier.

Researchers have assessed the performance of motile nanorobots in decontaminating intracellular ROS. One approach involved the development of hemin-loaded nanorobots based on mesoporous silica nanoparticles acting as ROS scavengers (Figure 8a) [66]. Hemin serves as the catalytic engine for decomposing ROS, generating random intracellular motion for nanorobots (Figure 8b). Such effective propulsion enhanced the ROS scavenging efficacy of nanorobots compared to static counterparts. Another strategy employed the classic catalytic engine, Pt, to design nanorobotic ROS scavengers [68]. This nanorobot was prepared by Janus-coating Pt on a silica surface (Figure 8c), followed by electrostatic absorption of black phosphorous quantum dots (BPQDs). Incorporated BPQDs enhanced the propulsion efficiency of the nanorobots compared to those without BPQDs, attributed to increased oxygen abundance in the presence of BPQDs (Figure 8d). Additionally, BPQDs possess intrinsic scavenging capabilities for eliminating superoxide radicals (O_2_^•−^) and hydroxyl radicals (•OH). These self-powered nanorobots demonstrated effective removal of overexpressed ROS within cells.

These recent advancements demonstrate the significant potential of autonomous nanorobots to serve as motile scavengers for the rapid elimination of toxic substances within cells. With a wide range of options for nanorobotic engines and surface functionalization, the scope of scavenging candidates can be expanded to include other toxins, such as reactive nitrogen species and heavy metals.

### 3.4. Photo-Based Therapy

The versatile building entities and surface modifications bestow the capability of nanorobots to leverage an external light source not only as the power engine but also as the therapeutic tool [30]. Photo-based therapy has emerged as an advanced approach in the biomedical community by using a remote light source with precise spatial control and tunable wavelength [125]. The interaction of light with biological tissues, cells, or photosensitive agents elicits specific physiological responses for disease treatment. For example, photothermal therapy (PTT) employs light-absorbing agents like nanoparticles to convert light energy into heat, inducing hyperthermia in targeted tissues and resulting in various therapeutic effects [126]. Another widely reported therapeutic modality, photodynamic therapy (PDT), relies on the photochemical reaction of photosensitizers [127]. Upon light irradiation, photosensitizers can convert oxygen molecules to ROS, causing damage to intracellular components and ultimately leading to cell apoptosis.

**Figure 9:**
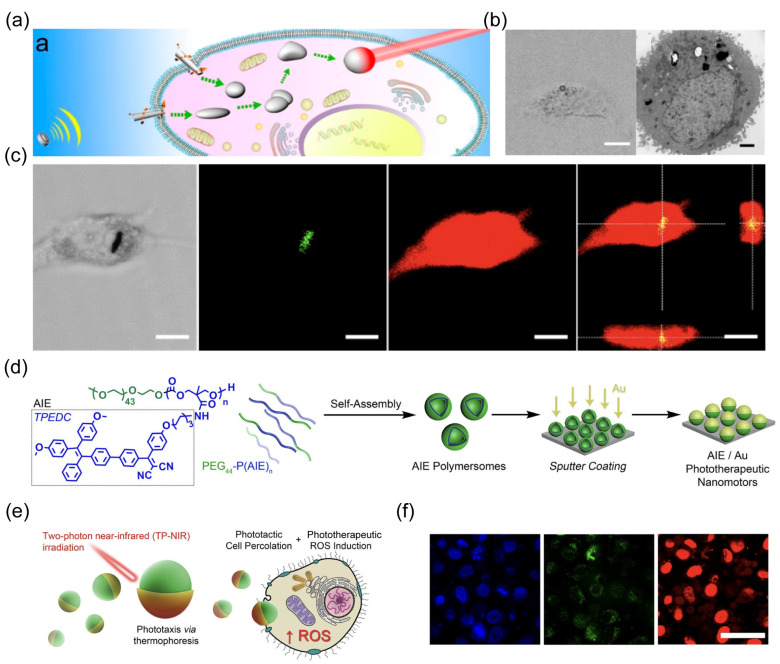
(**a**) Schematic showing that acoustically propelled liquid metal gallium nanorobots actively seek and target cancer cells, along with intracellular transformation, fusion, and photothermal cancer cell therapy. Reprinted with permission from [50]. Copyright © 2018, American Chemical Society. (**b**) CLSM image and TEM image of the HeLa cell containing the fused liquid metal droplets. Reprinted with permission from [50]. Copyright © 2018, American Chemical Society. (**c**) CLSM images illustrating the internalization of the nanorobot into a HeLa cell after 24 h. Scale bars, 10 μm. Reprinted with permission from [50]. Copyright © 2018, American Chemical Society. (**d**) Design of synergistic AIE-transduced phototherapeutic nanomotors. Reprinted with permission from [54]. Copyright © 2021, Springer Nature. (**e**) Schematic of NIR activation of nanorobots. Reprinted with permission from [54]. Copyright © 2021, Springer Nature. (**f**) Confocal images showing highly selective cell apoptosis using nanorobots with or without 200 s TP-NIR irradiation (nucleus: Hoechst, blue/viable cells: calcein-AM, green/apoptotic cells: PI, red). Scale bars, 50 μm. Reprinted with permission from [54]. Copyright © 2021, Springer Nature.

The small size and precise motion control of nanorobots enable rapid internalization by targeted cells [128]. Nanorobots can serve as photo-responsive agents to induce phototherapy at the cellular level, showing promising potential to maximize the therapeutic outcomes of phototherapy while minimizing unwanted side effects to normal tissues. Wang et al. presented a rodlike nanorobot composed of a liquid gallium core and solid gallium oxide shell (Figure 9a) [50]. These nanorobots exhibited robust movement upon an acoustic field due to the generated acoustic radiation force. The motility of nanorobots allows active seeking of targeted cells, leading to enhanced binding and penetration to the cells. Upon cellular internalization, the outer gallium oxide layer of nanorobots dissolved in acidic endosomes, resulting in shape transformation from the rod to droplet and subsequent fusion together (Figure 9b).

The inherent photothermal effect of liquid metal generated heat to kill cancer cells upon NIR illumination (Figure 9c). Cao et al. designed a nanorobot that employs light power as both a motion engine and a therapeutic tool [54]. The nanorobots were fabricated by asymmetrically coating self-assembled aggregation-induced emission (AIE) polymersomes with a Au shell **(**Figure 9d). The light propulsion of nanorobots induced by the photothermal effect of Au was enhanced by AIE polymersomes due to their capability of absorbing NIR for energy transduction **(**Figure 9e). Additionally, these AIE polymersomes can respond to NIR to generate ROS. The robust propulsion of nanorobots upon NIR irradiation enhanced their binding and percolation to targeted cells **(**Figure 9f). After entering cells, the intracellular ROS level was elevated to induce cell apoptosis due to the photodynamic feature of AIE polymersomes.

### 3.5. Drug Delivery

Traditional passive intracellular delivery commonly relies on natural endocytosis, which experiences a long period and low delivery efficiency. Motile nanorobots with enhanced intracellular internalization unlock an advanced biomedical vehicle to improve drug delivery into the cytoplasm, holding great potential for overcoming these limitations of passive delivery [38]. Considerable efforts have been devoted to designing nanorobotic platforms with various propulsion modes for adaptation to complex and practical biological environments [129]. The actuation mechanism can be mainly categorized into two types: chemical propulsion and actuation by external power sources.

**Figure 10:**
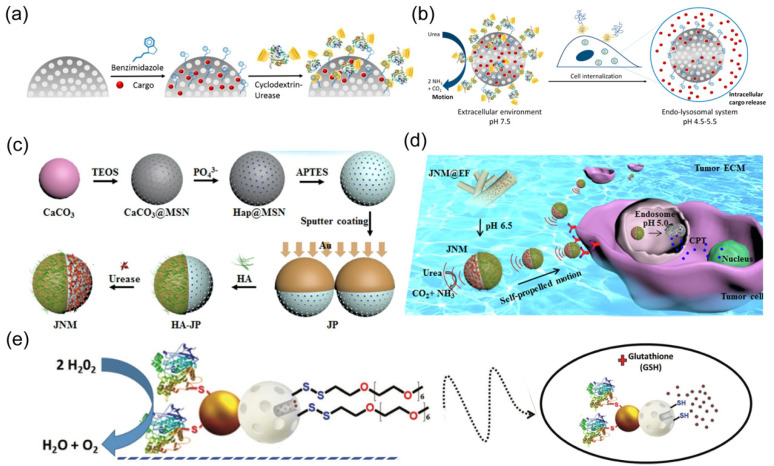
(**a**) Schematic of the fabrication process of urease-powered nanorobots. Reprinted with permission from [59]. Copyright © 2019, American Chemical Society. (**b**) The biocatalytic nanorobots exhibit effective propulsion and cargo release at acidic pH. Reprinted with permission from [59]. Copyright © 2019, American Chemical Society. (**c**) Fabrication process of urease-powered Janus nanorobot. Reprinted with permission from [60]. Copyright © 2019, Elsevier. (**d**) Schematic showing self-powered Janus nanorobot actively binding with targeted cells in tumor tissues. Reprinted with permission from [60]. Copyright © 2019, Elsevier. (**e**) Schematic of catalase-powered nanorobots with glutathione-responsive cargo delivery. Reprinted with permission from [64]. Copyright © 2019, Royal Society of Chemistry.

Regarding the chemical-powered nanorobots, ensuring the biocompatibility of the propulsive fuel is paramount for their successful deployment at the intracellular level [17]. Biocatalytic enzyme engines capable of harvesting propulsive energy from bioavailable fuels offer a feasible route to constructing biocompatible nanorobots that obviate the need for toxic fuels in early studies, such as H_2_O_2_. A urease-propelled mesoporous silica nanorobot was designed for active intracellular payload delivery with pH-responsive control (Figure 10a) [59].

This nanorobot was first modified with benzimidazole-functionalized cargo, followed by capping with cyclodextrin-modified urease. The formed complexes between benzimidazole and cyclodextrin-modified urease at neutral pH prevented cargo leakage of nanorobots due to the bulky caps. The acidic pH could trigger disassembly of the outer complexation to induce drug release (Figure 10b). The nanorobots exhibited effective propulsion in the presence of urea fuel, enhancing their internalization into cancer cells. The acidic lysosome facilitated the intracellular release of loaded drugs, such as [Ru(bpy)_3_]Cl_2_ or doxorubicin. Similarly, the urease engine was also employed to construct nanorobots for intracellular camptothecin (CPT) delivery [60]. In this design, hyaluronic acid (HA) and urease were modified on opposite sides of Janus nanorobots, serving as the targeting component and power source, respectively (Figure 10c). Given that H_2_O_2_ concentration is commonly elevated in lesion tissues or cells [130], this fuel can be leveraged by a catalase engine to provide sufficient driving force for nanorobots in biological environments (Figure 10d). Sun et al. engineered a stomatocyte polymersome nanorobot loaded with catalase [131]. The biocatalytic propulsion of nanorobots in the presence of H_2_O_2_ was verified to enhance their uptake by HeLa cells. Another catalase-powered nanorobot was prepared based on Janus Au–mesoporous silica nanoparticles (Au–MSNPs) (Figure 10e) [64]. Here, the silica surface was loaded with drug and subsequently immobilized with disulfide-linked oligo (ethylene glycol) (SS-OEG) chains acting as a responsive gate. This gate remained closed without stimulation but opened in the presence of glutathione (GSH) to trigger drug release. The effective motion of nanorobots in H_2_O_2_ solution was shown to enhance their cellular internalization and further induce drug release in the presence of intracellular GSH.

**Figure 11:**
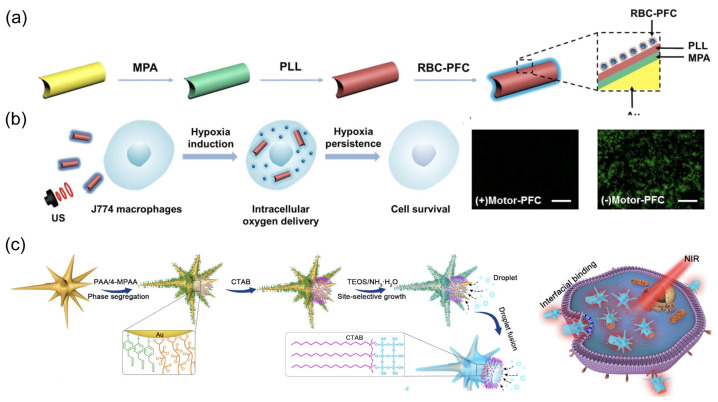
(**a**) The fabrication process for gold nanowire robots with red blood cell membrane-cloaked perfluorocarbon nano-emulsions (Motor-PFC). Reprinted with permission from [51]. Copyright © 2019, American Chemical Society. (**b**) Schematic and fluorescent images of nanorobot-based active O_2_ intracellular delivery system. Intracellular hypoxic stress is indicated by green fluorescence marker. Scale bars: 100 μm. Reprinted with permission from [51]. Copyright © 2019, American Chemical Society. (**c**) Schematic of the site-selective super-assembly process of nanorobots with urchin head/hollow tail nanostructures (UHHTN) and the process for triple-negative breast cancer (TNBC) treatment with UHHTN nanorobots. Reprinted with permission from [67]. Copyright © 2023, Springer Nature.

The utilization of external power sources for nanorobots also offers distinct advantages in enhancing their intracellular delivery [132]. Zhang et al. developed an acoustic-powered Au nanowire robot for intracellular oxygen delivery (Figure 11a) [51]. This nanorobot was surface-modified with red blood cell membrane-cloaked perfluorocarbon nanoemulsions (RBC-PFC) capable of high oxygen carrying. The efficient propulsion of nanorobots under an acoustic field augmented their cellular uptake and facilitated oxygen delivery to the cytoplasm, thereby maintaining cell viability under hypoxic conditions (Figure 11b). The Kong group reported a light-powered nanorobot with an urchin head and hollow tail (Figure 11c) [67]. The head region comprised a thin SiO_2_ shell with half-coating of Au nanostars, while the hollow tail allowed for the co-encapsulation of stimulus-responsive phase-change materials (PCMs) and doxorubicin (DOX). The release of PCMs and DOX could be triggered by the photothermal effect of Au under NIR irradiation. The biomimetic nanospike surface nano-topology, coupled with the active mobility of the nanorobots, worked synergistically to significantly enhance tumor penetration and cellular uptake for triple-negative breast cancer therapy.

### 3.6. Organelle Targeting

Cells are the basic unit of life for all life forms. Cellular activities are sustained and manipulated by the subsystem of organelles, such as the nucleus, lysosomes, mitochondrion, endoplasmic reticulum, and Golgi apparatus. The intracellular dynamics and functions of organelles regulate the metabolic status at the cellular level, further altering the homeostasis of living organisms. The organelle dysfunction can directly induce diverse diseases (e.g., cancer) [133]. Thus, treating organelles as the therapeutic target shows promising potential to improve the targeting and curative efficacy of prevalent pathologies [134]. However, the present organelle-level targeting heavily relies on passive targeting mechanisms by leveraging the intrinsic features of organelles, such as acidic lysosome lumen and high mitochondrial membrane potential. The motility deficiency of internalized cargo constrains their binding efficiency with specific organelles, leading to compromised benefits in clinical trials. Nanorobots capable of steerable motion in confined spaces are expected to introduce a new generation of robotic devices to achieve organelle targeting for precision therapy.

**Figure 12:**
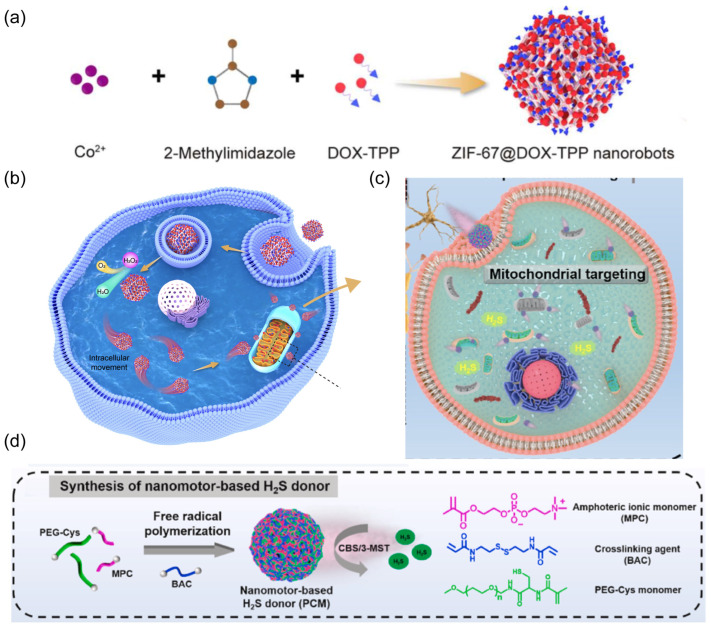
(**a**) Schematic of the fabrication of ZIF-67@DOX-TPP nanorobots. Reprinted with permission from [70]. Copyright © 2023, The American Association for the Advancement of Science. (**b**) The intracellular mitochondrial-targeted motion of nanorobots, enabling mitochondrial-targeted drug delivery to effectively inhibit cancer growth and metastasis. Reprinted with permission from [70]. Copyright © 2023, The American Association for the Advancement of Science. (**c**) Schematic of the treatment strategy for PD by nanorobots. (**d**) The synthetic process for nanorobot-based H_2_S donor PCM. Reprinted with permission from [71]. Copyright © 2024, Elsevier.

The Wu group fabricated mitochondrial-targeted nanorobots by encapsulating the mitochondriotropic drug doxorubicin-triphenylphosphonium (DOX-TPP) inside zeolitic imidazolate framework-67 (ZIF-67) nanoparticles (Figure 12a) [70].

The overexpressed H_2_O_2_ inside tumor cells was leveraged as propulsion fuel (Figure 12b). The catalytic ZIF-67, serving as the power engine, decomposed intracellular H_2_O_2_ to provide sufficient propulsive force for nanorobots in the cytoplasm. Mitochondria play a pivotal role in modulating cellular dynamic processes, such as calcium regulation, adenosine triphosphate (ATP) production, and cell apoptosis [135]. Mitochondria have been considered a subcellular therapeutic target because their dysfunction can result in a variety of pathologies, including inflammation, cancer growth and metastasis, and neurodegeneration [136]. The nanorobots exhibited effective propulsion inside tumor cells rather than normal cells due to the lack of sufficient H_2_O_2_ fuel. The loaded lipophilic and cationic TPP^+^ leads to mitochondrial-targeted propulsion for nanorobots, enhancing drug accumulation around mitochondria to damage and dysregulate this organelle. This active mitochondria-targeted behavior of nanorobots was demonstrated to upgrade the suppression of tumor growth and metastasis. In another work, mitochondrial-targeted nanorobots were prepared to act as H_2_S donors for the treatment of Parkinson’s disease (Figure 12c) [71]. The robotic body was constructed by a free radical polymerization reaction between polyethylene glycol (PEG) modified with L-cysteine derivative (PEG-Cys) and 2-methacryloyloxyethyl phosphorylcholine (MPC). Endogenous enzymes such as cystathionine β-synthase (CBS) in brain neurons or 3-mercaptopyruvate sulfurtransferase (3-MST) in mitochondria catalyzed the decomposition of L-cysteine, generating H_2_S to propel the nanorobots towards mitochondria within neural cells (Figure 12d). This active mitochondrial-targeted H_2_S delivery effectively eliminated ROS and alleviated the damage to neurons, thereby improving the therapeutic efficacy of Parkinson’s disease in a mouse model.

These works demonstrate that biocatalytic reactions between a robotic body and endogenous components create sufficient propulsive force to overcome constraints in narrow cytoplasm. The loaded chemotactic drug navigates these self-powered nanorobots toward targeted organelles with enhanced drug accumulation. The targeted organelle can be readily expanded from mitochondria to other organelles, such as endoplasmic reticulum and Golgi apparatus, for treating broader prevalent pathologies and diseases.

## 4. Conclusions and Outlook

In this review, we have first discussed four propulsion modes of nanorobots, identifying the propulsion mechanisms, robotic structures, and material selections involved in each mode. Detailed examples from previous works have been listed to show how the constituent materials react with the surrounding solution or respond to external power fields to obtain a robust driving force. Catalytic reactions between the robotic body and the surrounding fuel and Janus structure are essential to achieve chemical propulsion. The Au nanorod robot with a concave end is the most prevalently used building entity for acoustic-powered nanorobots. Bacterial flagella inspire the use of helical structure and a soft tail with magnetic layer coating, enabling effective motion in response to a rotating magnetic field. Light propulsion requires the incorporation of photochemical or photothermal materials into the robotic body. Next, we identified six main aspects of intracellular applications, illustrating the material and propulsion choices and highlighting the advantages of nanorobots in each context. The effective and steerable motion of nanorobots can overcome the constraints in complicated biological environments to actively target and reach desired sites for accomplishing biomedical missions. These tiny machines can open the cell membrane with the assistance of an external power source. Their robust locomotion enables accelerated cellular internalization to induce rapid transport of biosensor probes and therapeutic cargo into the cytoplasm. Additionally, the internalized nanorobots can serve as toxin scavengers or organelle-targeted vehicles to directly modulate cell metastasis and organelle functions. The autonomous nanorobots introduce a new generation of active medical tools with expanded operation scope in the cellular or subcellular level. The unlocked profound dimension enables nanorobots to act as miniaturized surgeons as envisioned in science fiction movies to specifically target and regulate cellular dynamics and metabolism for precision therapy.

**Figure 13:**
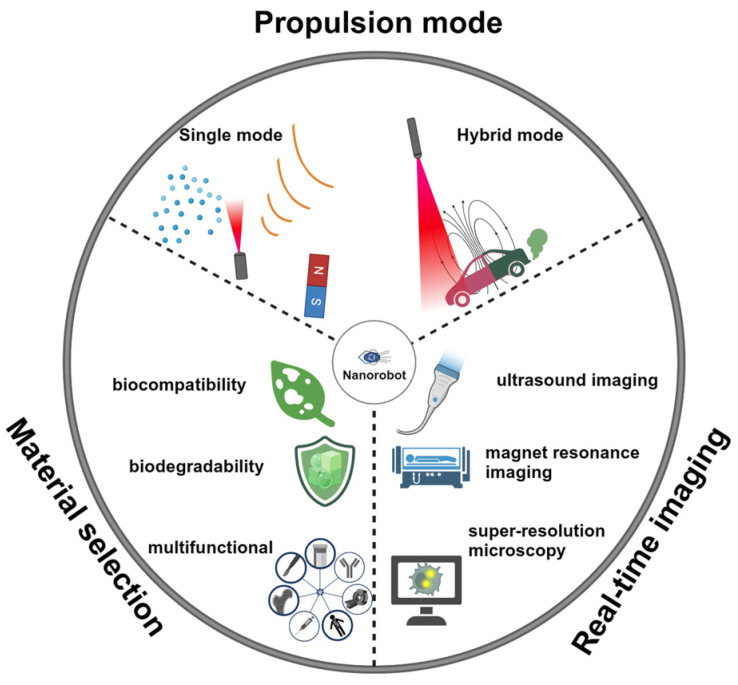
Potential research directions for future nanorobot development in intracellular applications.

Despite these considerable efforts devoted by worldwide researchers, the development of nanorobots at cellular-level operations is still in the early stages. The current challenge mainly lies in the following aspects: (1) Improving the biocompatibility and biodegradability of nanorobots; (2) achieving effective intracellular propulsion with simplified equipment and without introducing toxic substrates; and (3) real-time visualization of nanorobot operations inside cells. Here, we discuss three points to propose potential solutions for addressing these challenges (Figure 13), aiming to suggest research directions for future nanorobot development in intracellular applications.

**(1). Material Selection**

Traditional metal or inorganic materials, such as Pt or SiO_2_, cannot meet the rigorous biosafety requirements of practical biomedical applications. Biocompatible and biodegradable materials are highly preferable in building nanorobots for intracellular applications. Additionally, exogenous counterparts may evoke immune clearance and cellular dysregulation. Endogenous components emerge as ideal candidates for constructing nanorobots, offering inherent biocompatibility, biodegradability, and biological functionality, including cell and organelle targeting. An illustrative example is coating nanorobots with cell membranes, which enhances their biocompatibility and functionality.

**(2). Propulsion Mode**

Chemical propulsion obviates the requirement of an external field, avoiding the complexity associated with actuation equipment. The employment of biocatalytic engines with coordinated bioavailable fuel is expected to fabricate a fully biocompatible nanorobot. Enzyme engines, such as catalase, urease, and Gox, are attractive options. However, the chemical motion of nanorobots is usually defined as enhanced diffusion, whose robustness may not be comparable with an external power source. The potential toxicity and tissue penetration depth are the main considerations when using external power sources. Meanwhile, a simplified actuation system is crucial to lowering the barriers to propelling nanorobots. Biological propulsion that utilizes the motility of intracellular motile proteins or molecules is another promising approach to designing self-powered nanorobots for intracellular applications [137]. This propulsion mode is expected to leverage the cellular machinery energy to propel nanorobots. On the other hand, the combination of various propulsion modes offers a promising avenue to enhance motion performance and the adaptation of nanorobots in dynamic and complex environments [138]. These hybrid-powered nanorobots capable of controllable propulsion behavior, such as speed up or down, directional reversal, or shape reconfiguration, hold considerable potential to expand their versatility in practical intracellular applications.

**(3). Real-Time Imaging**

The imaging tool is essential to visualize and track the movement, localization, and distribution of nanorobots within live cells. This enables the assessment of cellular uptake, intracellular trafficking pathways, and subcellular targeting of nanorobots with high spatial and temporal resolution. Various imaging tools can be considered potential candidates, such as photoacoustic (PA) imaging, ultrasound imaging, magnet resonance imaging (MRI), fluorescence microscopy, confocal microscopy, two-photon microscopy, and super-resolution microscopy. The use of imaging tools requires consideration of the small size of nanorobots and the tissue penetration depth for future in vivo applications. Real-time imaging enables feedback-guided control and manipulation of nanorobots within live cells, allowing researchers to dynamically adjust experimental parameters, interventions, or stimuli based on observed responses or outcomes. This closed-loop feedback system enhances the precision and effectiveness of intracellular nanorobot applications.

## Figures and Tables

**Table 1:** Summary of representative nanorobots for intracellular applications.

Materials	Size	Propulsion Mechanism	Application	Ref.
AuNW	4 μm in length and 200 nm in diameter	US	biosensing	[45]
AuNW	1.7 μm in length and 400 nm in diameter	US	biosensing	[46]
AuNW	2 μm in length and 400 nm in diameter	US	cargo delivery	[47]
AuNW	4 μm in length and 200 nm in diameter	US	cargo delivery	[48]
AuNW	4 μm in length and 200 nm in diameter	US	cargo delivery	[49]
gallium	5.5 μm in length and 500 nm in diameter	US	photo-based therapy	[50]
RBC-PFC, AuNW,	2 μm in length and 400 nm in diameter	US	cargo delivery	[51]
gold, polymer	10 pm in length, diameters of the two openings are ~200 nm and ~800 nm	US	cell membrane penetration	[52]
Au/Ni/Si	~5 μm in length with tip diameters < 50 nm	MF	cargo delivery	[53]
polymersomes, Au	around 400 nm	NIR	biosensing	[54]
SiO_2_-Co/Fe	2.4 μm in length and 250 nm in width	MF	biosensing	[55]
Ni-carbon	<2 μm in length	MF	cargo delivery	[56]
Ni/Pt/Ni	200 nm in diameter and 1.5 mm in length	MF	cargo delivery	[57]
Pt, polymer	500 nm	H_2_O_2_	cargo delivery	[58]
mesoporous silica NPs	average diameter ~420 nm	urea	cargo delivery	[59]
mesoporous silica NPs, gold	average diameter of sub-100 nm	urea	cargo delivery	[60]
carbon, Fe_3_O_4_	outer diameter of 10–15 nm, length of 1–5 μm	MF	cargo delivery	[61]
cetyltrimethylammonium bromide (CTAB) and tetraethylorthosilicate (TEOS)	average diameter 344 ± 3 nm	urease	cargo delivery	[62]
gold	average diameter 171.53 + 1.40 nm	H_2_O_2_	cargo delivery	[63]
AuNW, red blood cell membrane	2 μm in length and 400 nm in diameter.	US	cargo delivery	[51]
Au-mesoporous silica	Au 20nm, SiO_2_ 80 nm	H_2_O_2_	cargo delivery	[64]
mesoporous silica	418 ± 21 nm	urea	cargo delivery	[59]
Yb mof	41 ± 2 nm	GOx-Cat	cargo delivery	[65]
mesoporous silica nanoparticles, hemin	diameter of about 630 nm	ROS	detoxification	[66]
AuNS, SiO_2_	length (~13–94 nm), tail length (~0–510 nm), and large tunable hollow diameter (~100–240 nm)	NIR	cargo delivery	[67]
Au, MnO_2_	93.4 nm	NIR	biosensing	[28]
polymersomes, Au	around 400 nm	NIR	photo-based therapy	[54]
Pt, silica, black phosphorous	diameter (450 nm)	H_2_O_2_	detoxification	[68]
calcium carbonate nanoparticle	a diameter of 60.0 ± 5.0 nm	H_2_O_2_	cargo delivery	[69]
ZIF-67, DOX-TPP	average size of 140.0 nm	H_2_O_2_	organelle targeting	[70]
PEG-Cys, MP, PEG	average size 200 nm	H_2_S	organelle targeting	[71]
mesoporous silica	67.8 nm to 80.6 nm	NIR	cell membrane penetration	[72]
gold nanowire	2 μm in length and 400 nm in diameter	US	biosensing	[73]
